# Linalool Alleviates A*β*42-Induced Neurodegeneration via Suppressing ROS Production and Inflammation in Fly and Rat Models of Alzheimer's Disease

**DOI:** 10.1155/2021/8887716

**Published:** 2021-03-10

**Authors:** Chunyu Yuan, Myeongcheol Shin, Youngjae Park, Byoungyun Choi, Seokhui Jang, Chaejin Lim, Hye Sup Yun, Im-Soon Lee, So-Yoon Won, Kyoung Sang Cho

**Affiliations:** ^1^Department of Biological Sciences, Konkuk University, Seoul 05029, Republic of Korea; ^2^CHANS Research Center, Konkuk University, Seoul 05029, Republic of Korea; ^3^Korea Hemp Institute, Konkuk University, Seoul 05029, Republic of Korea

## Abstract

Terpenes are vital metabolites found in various plants and animals and known to be beneficial in the treatment of various diseases. Previously, our group identified terpenes that increased the survival of Alzheimer's disease (AD) model flies expressing human amyloid *β* (A*β*) and identified linalool as a neuroprotective terpene against A*β* toxicity. Linalool is a monoterpene that is commonly present as a constituent in essential oils from aromatic plants and is known to have anti-inflammatory, anticancer, antihyperlipidemia, antibacterial, and neuroprotective properties. Although several studies have shown the beneficial effect of linalool in AD animal models, the mechanisms underlying the beneficial effect of linalool on AD are yet to be elucidated. In the present study, we showed that linalool intake increased the survival of the AD model flies during development in a dose-dependent manner, while the survival of wild-type flies was not affected even at high linalool concentrations. Linalool also decreases A*β*-induced apoptosis in eye discs as well as the larval brain. Moreover, linalool intake was found to reduce neurodegeneration in the brain of adult AD model flies. However, linalool did not affect the total amount of A*β*42 protein or A*β*42 aggregation. Rather, linalool decreased A*β*-induced ROS levels, oxidative stress, and inflammatory response in the brains of AD model flies. Furthermore, linalool attenuated the induction of oxidative stress and gliosis by A*β*_1-42_ treatment in the rat hippocampus. Taken together, our data suggest that linalool exerts its beneficial effects on AD by reducing A*β*42-induced oxidative stress and inflammatory reactions.

## 1. Introduction

Alzheimer's disease (AD) refers to a neurodegenerative condition, which is recognized as the most common cause of dementia [1]. The hallmarks of AD are abnormal aggregation of amyloid *β* (A*β*) and tau protein, and the accumulation of the pathological forms of these proteins has been considered to be a cause of AD [2]. In particular, the accumulated A*β* forms toxic oligomers, which subsequently induce various pathophysiological events such as inflammation, reactive oxygen species (ROS) generation, and neuronal death during the progression of AD [3–6].

Currently, four drugs, including donepezil, galantamine, rivastigmine, and memantine, have been approved by the U.S. Food and Drug Administration for the treatment of AD [7]. However, these treatments are used as temporary memory enhancers to relieve symptoms rather than to modify disease progression. In addition, these treatments are associated with adverse effects related to the gastrointestinal tract and cardiovascular system [8, 9]. Therefore, studies on the neuroprotective effects of natural compounds that do not exhibit any pronounced toxicity are being conducted to replace the current treatments for AD.

Terpenes are natural compounds that have a potential to treat AD. They are volatile organic compounds that contain hydrocarbons with 5 carbon atoms as their building blocks. Terpenes are produced by various organisms, especially plants [10, 11]. Previous studies have shown that many terpenes have anti-inflammatory, antitumor, or neuroprotective properties [12]. Based on that, our prior screening identified terpenes that exhibit neuroprotective effects against A*β* cytotoxicity using a *Drosophila* AD model and identified six terpenes, namely, *ρ*-cymene, limonene (+), limonene (-), linalool, *α*-pinene (+), and *β*-pinene (-), as neuroprotective terpenes that can effectively suppress the A*β* phenotype in AD flies [13].

Linalool is a monoterpene that is commonly present as a constituent in essential oils from aromatic plants. Linalool is known to have anticancer, antihyperlipidemia, antibacterial, and neuroprotective properties and has a wide range of biological activities, including antioxidant and anti-inflammatory effects [14, 15]. It is a colorless and volatile monoterpene found in essential oils from more than 200 plants [16, 17]. Several studies have shown that linalool exerts a neuroprotective effect by inhibiting inflammation *in vitro* and *in vivo* [18–20]. Linalool also protects neurons by decreasing ROS levels in patients with carpal tunnel syndrome, which is a ROS-induced peripheral neuropathy [21]. Importantly, the safety of linalool is attested by the fact that it has been approved as a food flavoring by the European Commission [22]. Therefore, linalool is a promising natural compound for treating diseases related to ROS and inflammation [14, 15].

The beneficial effects of linalool on neurodegeneration were reported in two AD mouse models. Linalool reduced A*β* aggregation in a transgenic AD mouse model and reversed A*β*-induced memory loss in both transgenic and A*β*-injected AD mouse models [23, 24]. However, the detailed molecular mechanisms by which linalool protects the neurons from A*β* toxicity are not fully understood. Therefore, the use of linalool as a treatment for AD needs to be studied in many respects.

In the present study, we showed that linalool intake suppressed A*β*-induced neuronal cell death without affecting A*β* aggregation in the brains of flies expressing human A*β*. Linalool ameliorated AD-like phenotypes, including neurodegeneration, through its anti-inflammatory and antioxidative properties in fly and rat AD models. These results suggest that linalool is a potential therapeutic agent for AD treatment.

## 2. Materials and Methods

### 2.1. *Drosophila* Strains

The *w*^1118^, glass multimer reporter-GAL4 (*GMR-GAL4*; eye driver), embryonic lethal abnormal vision-GAL4 (*elav-GAL4*; pan-neuronal driver), and Drosomycin-tagged green fluorescent protein (*Drs-GFP*) strains were obtained from the Bloomington *Drosophila* Stock Center (Bloomington, IN, USA). The *UAS-Aβ42*^2X^ strain was a gift from Dr. Fernandez-Funez (University of Florida, USA). The genotypes of the flies were *elav>Aβ42*^2X^ (*elav-GAL4/+*; *UAS-Aβ42*^2X^*/+*) and *GMR>Aβ42*^2X^ (*GMR-GAL4*, *UAS-Aβ42*^2X^*/+*).

### 2.2. Survival Assay

In order to determine the survival rate of *Drosophila*, 250 *Drosophila* embryos were collected on grape juice agar plates. Every 50 embryos were transferred into a vertical plastic vial and maintained at 25°C and 60% humidity. The survival rate of the female adults was estimated. DMSO-fed *w*^1118^ and DMSO-fed *elav>Aβ42*^2X^ were used as controls. This experiment was repeated three times (total of 750 embryos per group were used).

### 2.3. Acridine Orange Staining

Acridine orange (AO) staining was performed to detect cell death. As previously described [13], the brain or eye discs of L3 larvae were dissected in phosphate buffer saline (PBS, pH 7.8, Bio Basic, Seoul, Republic of Korea) and incubated with 1.6 × 10^−6^ M AO (Sigma Aldrich, St. Louis, USA) for 5 min at room temperature. The samples were washed twice with PBS for 5 min each. The cells were examined under an Axiophot 2 fluorescence microscope (Carl Zeiss, Oberkochen, Germany), and then the number of stained cells was counted.

### 2.4. Brain Section

To measure neuronal loss, the fly heads were sectioned as previously reported [25]. Fly heads were fixed in Carnoy solution (60% ethanol, 30% chloroform, and 10% glacial acetic acid) at 4°C for 4 d and embedded in paraffin. The embedded heads were sectioned (5 *μ*m thick sections) and stained with hematoxylin and eosin. The stained samples were examined under a light microscope.

### 2.5. Immunohistochemical Analysis of *Drosophila* Brain

Fly brains were fixed in 4% paraformaldehyde (PFA) prepared in PBST (PBS + 0.5% Triton X‐100) for 3 h at room temperature. The samples were washed thrice with PBST for 10 min and incubated in blocking solution (2% normal goat serum + 2% bovine serum albumin (BSA) + 0.5% Triton X-100) for 3 h. Then, the samples were incubated with anti-A*β*42 antibody prepared in the blocking solution for 48 h at 4°C (blocking solution 1 : 200; Santa Cruz, CA, USA). Alexa Fluor 555 anti-rabbit antibody (PBST 1 : 200; Cell Signaling Technology, Danvers, USA) was used as the secondary antibody.

### 2.6. Measurement of ROS Levels

The ROS levels in the eye discs were measured using dihydroethidium (DHE; Invitrogen molecular probe, USA). As previously described [13], the eye discs of L3 larvae were dissected and incubated with Schneider's medium containing 40 *μ*M DHE for 5 min and then washed twice in Schneider's medium for 5 min each time. The samples were observed under an Axiophot 2 fluorescence microscope (Carl Zeiss).

### 2.7. Measurement of Nitric Oxide Levels

As previously described [13], the heads of 3- to 5-day-old flies (*n* = 15) were resuspended in 10× PBS on ice. The samples were ground and centrifuged at 10,000 × *g* for 10 min at 4°C. The supernatants were mixed with Greiss reagent at 1 : 1 ratio (Sigma-Aldrich) and incubated for 15 min at 25°C. Nitrite oxide (NO) levels were measured at 550 nm using a spectrophotometer.

### 2.8. Measurement of Inflammation after Bacterial Infection

The DH5*α* strain of *Escherichia coli* (Dongin Biotech, Republic of Korea) was cultured in Luria Bertani medium at 37°C for 16–20 h and concentrated by centrifugation. Septic injury was induced by pricking the thorax of 3- to 5-day-old *Drs-GFP* adult females with a thin needle dipped into a concentrated bacterial pellet (4.4 × 10^10^ cfu/ml) [13].

### 2.9. Animals

All experimental procedures were in accordance with the guidelines of the Laboratory Animal Manual of the National Institutes of Health Guide to the Care and Use of Animals, which were approved by the Ethics Review Committee of Konkuk University for Animal Experiments (approval number: KU20170). Sprague-Dawley rats, 260 to 280 g at the time of surgery, were housed two to three per cage with ad libitum access to water and food during a 12-hour light/dark cycle.

### 2.10. Preparation of A*β* Peptide

A*β*_1-42_ (Invitrogen, Camarillo, CA) were prepared as described previously [26] by dissolving the peptide in a freshly prepared 35% acetonitrile solution with dilution to a final concentration of 0.5 mM in PBS (pH 7.4). The A*β*_1-42_ solution was incubated for 24 h at 37°C to prepare fibrillary aggregates. After incubation, A*β*_1-42_ were stored at -20°C until use.

### 2.11. Stereotaxic Surgery

Rats were anesthetized and positioned in a stereotaxic apparatus (David Kopf Instruments, Tujunga, CA). A midline sagittal incision was made in the scalp, and holes were drilled in the skull over the dorsal hippocampus using the following coordinates: 3.6 mm posterior to the bregma and 2.0 mm lateral to the midline for intrahippocampal injections according to the atlas of Paxinos and Watson [27]. The hole of the tip was directed down to 2.6 mm beneath the surface of the brain for the hippocampus. All injections were made using a Hamilton syringe equipped with a 30S gauge beveled needle and attached to a syringe pump (KD Scientific, New Hope, PA). Infusions were made at a rate of 0.2 *μ*l/min for A*β*_1-42_ (1 nmol in 2 *μ*l). After injection, the needle was left in place for an additional 5 min before being slowly retracted.

### 2.12. Linalool Injection

Linalool was administered intraperitoneally (i.p.) once a day for 7 days before surgery and 14 days after surgery. Linalool was dissolved in DMSO (vehicle) and injected at doses of 50 and 100 mg/kg. The control groups received the same volume of vehicle for 3 weeks.

### 2.13. Rat Brain Tissue Preparation and Immunostaining

Rats were anesthetized at the indicated time points after injection and were perfused transcardially with a 0.9% saline solution containing 0.5% sodium nitrate and heparin (10 U/ml), which was followed by fresh cold 4% PFA fixative (pH 7.4). Brains were removed from the skull, postfixed in 4% PFA for 24 h at 4°C, and then placed in a 30% sucrose solution until they sank. Brains were frozen sectioned using a sliding microtome into 35 *μ*m coronal sections after they were embedded in optimum cutting temperature compound (Surgipath), and they were then collected into six separate series. Sections were stored free-floating in cryopreservative medium at −20°C. The tissue sections were washed in cold PBS for 15 min and incubated with a universal blocking solution in PBS (0.3% Triton X-100, 1% BSA, 0.05% Tween 20, 0.1% cold fish gelatin, and 0.05% sodium azide) for 1 h at room temperature. Brain sections were rinsed in PBS then incubated with the following primary antibodies: antiglial fibrillary acidic protein (GFAP, 1 : 500, mouse, Sigma Aldrich) for astrocytes and anti-4-hydroxynonenal (4-HNE, 1 : 300, mouse, abcam, Cambridge, UK) for lipid peroxidation. For fluorescence microscopy, the samples were incubated with Alexa 594-conjugated secondary antibodies (Cell Signaling Technology). For light microscopy, brain tissues were incubated with a biotin-conjugated secondary antibody followed by streptavidin-conjugated HRP (Vectastain ABC kit, Vector Laboratories). Immunostaining was visualized by incubating the samples in a 0.1 M-PB solution containing 0.05% diaminobenzidine-HCl and 0.003% hydrogen peroxide.

### 2.14. Statistical Analysis

Data were quantitatively analyzed for significance using either Student's *t*-test (two-tailed) or one-way ANOVA followed by Tukey-Kramer multiple comparison test. Student's *t*-test was used for comparisons between two groups. GraphPad Prism 8.0 (GraphPad Software Inc., USA) was used for performing statistical analyses. *P* values < 0.05 indicated a significant difference.

## 3. Results

### 3.1. Linalool Intake Increases Survival during the Development of the *Drosophila* AD Model

**Figure 1:**
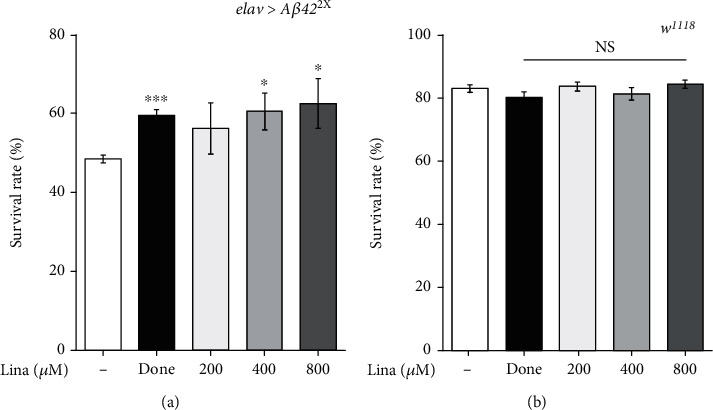
Linalool intake increases survival of the *Drosophila* AD model during development. The graphs show the effect of linalool on the survival of *Drosophila* expressing *Aβ42* (*elav>Aβ42*^2X^) (a) and *w*^1118^ (b) during development (*n* = 750). Flies fed with donepezil (Done) were used as positive control. All data in the graphs are expressed as mean ± standard error of mean (SEM). ^∗∗∗^*P* < 0.001, ^∗^*P* < 0.05 vs. DMSO-fed *elav>Aβ42*^2X^ (a) or DMSO-fed *w*^1118^ (b); Student *t*-test. Done: 0.1 *μ*g/ml donepezil; Lina: linalool; NS: not significant.

It is known that the *Drosophila* AD model used in this study shows decreased survival compared to the wild-type *Drosophila* during development [13]. To confirm the effect of linalool on AD, we used the *Drosophila* AD model (*elav-GAL4>UAS-Aβ42*^2X^) that expresses *Aβ42* in the neurons. Donepezil (Done), a current AD treatment, was used as a positive control (Figure 1(a)). As expected, donepezil increased the survival of *Aβ42*-expressing flies during development (Figure 1(a)). Linalool also showed protective effects against A*β*42 toxicity in a dose-dependent manner. More importantly, linalool did not affect the survival of wild-type flies at the concentrations used in AD model flies (Figure 1(b)). These results demonstrate that linalool has a protective effect against A*β* toxicity and has no apparent harmful effects even at high concentrations.

### 3.2. Linalool Intake Suppressed A*β*42-Induced Cell Death

**Figure 2:**
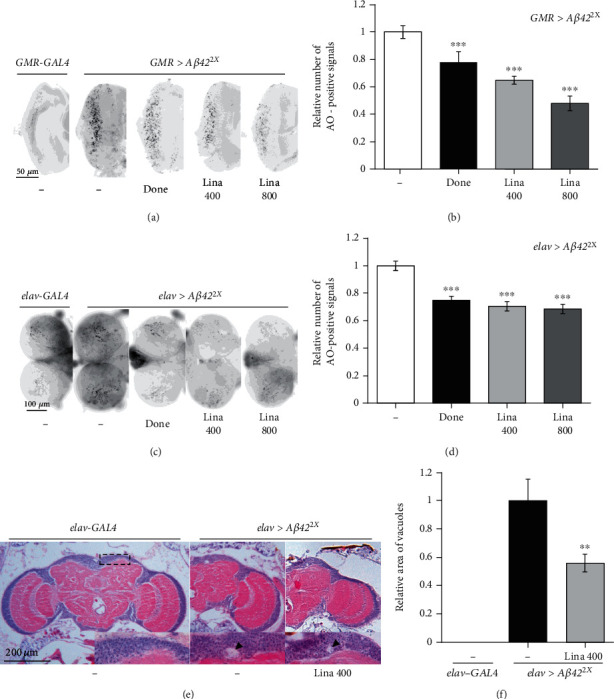
Linalool intake suppresses A*β*42-induced cell death and neurodegeneration. Linalool inhibited cell death in the *Drosophila* eye discs (a, b) and the larval brains (c, d) expressing *Aβ42*. (a, c) Representative images of acridine orange- (AO-) stained larval eye discs (a) and brains (c) of indicated groups. (b, d) The graphs show the relative number of AO-positive signals in the larval eye discs and brains of indicated groups (*n* ≥ 20). All data in the graphs are expressed as mean ± SEM. ^∗∗∗^*P* < 0.001 vs. DMSO-fed *elav>Aβ42*^2X^ (b) or DMSO-fed *GMR>Aβ42*^2X^ (d); Student's *t*-test. (e, f) Linalool inhibited neurodegeneration in the brains expressing *Aβ42*. (e) Representative optical microscopic images of the adult brains of control (*elav-GAL4*) and the AD model (*elav>Aβ42*^2X^). The dotted box is the region in which vacuoles were observed. Insets are magnified views of dorsomedial region of the brain in which vacuoles appeared. Arrowheads indicate vacuoles caused by neurodegeneration. (f) The graph shows the relative size of vacuole areas (*n* ≥ 7). All data in the graphs are expressed as mean ± SEM. ^∗∗^*P* < 0.01 vs. DMSO-fed *elav>Aβ42*^2X^ (f); Tukey–Kramer test. Done: 0.1 *μ*g/ml donepezil; Lina 400: 400 *μ*M linalool; Lina 800: 800 *μ*M linalool.

As linalool reduced A*β* toxicity during development, we investigated whether linalool intake suppressed A*β*42-induced cell death in the developing eyes and the brain using AO staining, which detects dead cells. Similar to a previous report [13], ectopic expression of human *Aβ42* in the developing eyes using *GMR-GAL4* driver (*GMR*>*Aβ42*^2X^) strongly induced cell death (Figures 2(a) and 2(b)). Linalool and donepezil significantly reduced A*β*42-induced cell death in the developing eye discs (Figures 2(a) and 2(b)). Consistently, linalool and donepezil treatment also suppressed A*β*42-induced cell death in the larval brain (Figures 2(c) and 2(d)). We also examined whether linalool intake suppresses neurodegeneration in the brains of *Aβ42*-expressing flies. Neurodegeneration leads to vacuolation in the brains of AD model flies [28], which is not observed in the brains of wild-type flies (Figure 2(e)). The vacuole area in the brains of flies fed with linalool was significantly decreased compared to that in the brains of control (DMSO-fed *elav*>*Aβ42*^2X^) flies (Figures 2(e) and 2(f)), demonstrating that feeding with linalool reduced neurodegeneration. Collectively, our results suggest that linalool protects neurons from neurodegeneration by inhibiting neuronal cell death in AD model flies.

### 3.3. Linalool Did Not Affect A*β*42 Protein Levels or Aggregation

**Figure 3:**
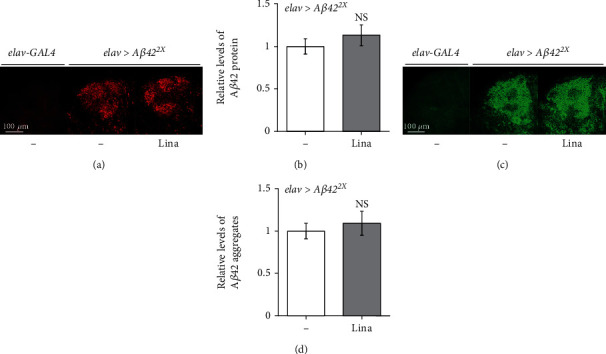
Linalool intake does not affect A*β*42 peptide accumulation and aggregation. The levels of A*β*42 peptide (a, b) and aggregation (c, d) in the *Drosophila* brains expressing *Aβ42* (*elav>Aβ42*^2X^) were not altered by linalool intake. (a, c) Representative confocal images of the Kenyon cell body region in the adult fly brains. The A*β*42 protein accumulation (a) and aggregation (c) in the brains of flies fed with linalool (*elav>Aβ42*^2X^, Lina) were compared to those in the brains of control (*elav>Aβ42*^2X^, DMSO) flies. Adult brains were stained with anti-A*β*42 antibody (a) or thioflavin S (c). (b, d) The graphs show the relative intensity of A*β*42-antibody staining (b) and thioflavin S staining (d) (*n* ≥ 20). All data in the graphs are expressed as mean ± SEM; Student's *t*-test. Lina: 400 *μ*M linalool; NS: not significant.

As A*β*42 aggregation and deposition are closely related to neuronal cell death, we investigated whether the inhibitory effect of linalool on cell death is due to a decrease in the amount of A*β*42 deposition or aggregation. Therefore, we compared the amount of A*β*42 protein accumulation in the brains of AD flies fed with linalool to that in brains of DMSO-fed control flies. The accumulated A*β*42 was visualized using immunohistochemistry. As shown in Figures 3(a) and 3(b), linalool intake did not alter A*β*42 levels in the brains of AD flies. We also examined the levels of A*β*42 aggregates by thioflavin S staining, which stains oligomeric fibrils or plaques of A*β*42 [29]. Similar to accumulated A*β*42 levels, aggregation of A*β*42 was not altered by linalool (Figures 3(c) and 3(d)). These results indicate that the neuroprotective effect of linalool is not achieved by inhibiting A*β*42 accumulation or aggregation.

### 3.4. Linalool Decreased A*β*42-Induced ROS Generation

**Figure 4:**
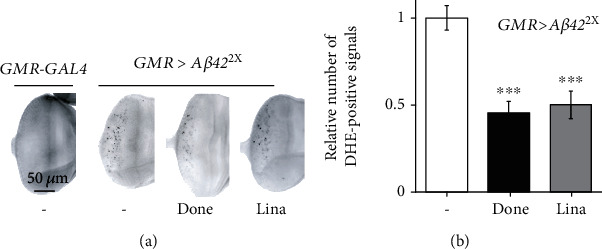
Linalool intake decreases ROS levels in the eye discs expressing *Aβ42*. (a) Representative DHE-staining images of larval eye discs expressing *Aβ42* (*GMR>Aβ42*^2X^). DHE-positive signals in the eye discs of linalool-fed flies were compared to those in the eye discs of control (*GMR>Aβ42*^2X^, DMSO fed) flies. (b) The graph shows the relative number of DHE-positive signals (*n* ≥ 10). All data in the graph are expressed as mean ± SEM. ^∗∗∗^*P* < 0.001 vs. DMSO-fed *GMR>Aβ42*^2X^ (b); Student's *t*-test. Done: 0.1 *μ*g/ml donepezil; Lina: 400 *μ*M linalool.

Given that linalool has an antioxidant effect [12], and that ROS is an important mediator of A*β*42 toxicity [30], ROS was measured by DHE staining to determine whether linalool prevents ROS production in AD flies through its antioxidant activity. Similar to a previous report [13], ectopic expression of *Aβ42* in the developing eyes (*GMR*>*Aβ42*^2X^) clearly induced ROS levels, as detected by DHE staining, while DHE-positive signals were not observed in control flies without *Aβ42* (*GMR*-*GAL4*) (Figure 4). Linalool and donepezil significantly decreased ROS levels (Figure 4), suggesting that the protective effects of linalool against A*β*42 toxicity are related to its antioxidant effect, at least in part.

### 3.5. Linalool Decreased Inflammation Induced by A*β*42 and Bacterial Infection

**Figure 5:**
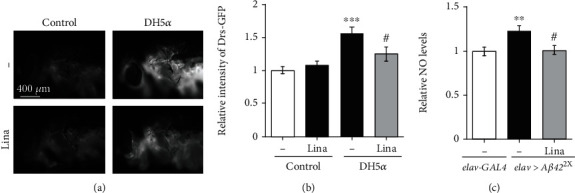
Linalool intake reduces inflammation induced by bacterial infection or *Aβ42* expression. (a) Fluorescent microscopic images of linalool- or DMSO-fed Drs-GFP flies that express *GFP* controlled by the *Drs* promoter. Sucrose is used for a negative control. (b) The graph shows the relative levels of GFP in the thorax of flies injected with sucrose or DH5*α* (*n* ≥ 10). (c) The effect of linalool intake on the elevated NO levels in *Aβ42*-expressing fly heads. The graph shows relative NO levels in the heads of adult flies (*n* ≥ 9). NO levels in the heads of flies expressing *Aβ42* (*elav>Aβ42*^2X^) were compared to those in the heads of control (*elav-GAL4*) flies. All data in the graphs are expressed as mean ± SEM. ^∗∗∗^*P* < 0.001, ^∗∗^*P* < 0.01 vs. DMSO-fed *elav-GAL4* (b) or DMSO-fed Drs-GFP injected with sucrose (c), ^#^*P* < 0.05 vs. DMSO-fed *elav>Aβ42*^2X^ (b) or DMSO-fed Drs-GFP injected with DH5*α* (c); Tukey–Kramer test. Lina: 400 *μ*M linalool; Drs-GFP: GFP-tagged Drosomycin.

Previous studies have shown that neuroinflammation plays a key role in the pathology of AD [5]. As linalool has been reported to exert anti-inflammatory effects [31, 32], we investigated whether the beneficial effect of linalool is owing to its suppressive function in inflammation. First, to examine whether linalool can reduce inflammation in *Drosophila*, we used a microbial infection model, in which inflammatory response can be measured by an antimicrobial peptide reporter system after septic injury that infects the microorganism. In this system, the levels of inflammatory response can be measured based on the expression levels of GFP-labeled Drosomycin (Drs-GFP) in *Drosophila*. As expected, GFP levels increased in the thorax of *Drosophila* following DH5*α* infection when compared to those in the thorax of sucrose-injected control *Drosophila* (Figures 5(a) and 5(b)). Interestingly, linalool intake significantly reduced the expression of DH5*α* infection-induced Drs-GFP (Figures 5(a) and 5(b)). Then, we assessed whether linalool intake reduced the inflammation caused by A*β*42 by measuring NO levels in the fly brains. Similar to a previous report [13], *Aβ42* expression in neurons increased NO levels in the brain of flies (Figure 5(c)). Moreover, linalool decreased NO levels in the AD flies to the levels of the control flies (Figure 5(c)). These results indicate that linalool suppresses inflammation in AD flies through its anti-inflammatory effect.

### 3.6. Linalool Reduced A*β*_1-42_-Induced Oxidative Stress and Gliosis in the Rat Hippocampus

**Figure 6:**
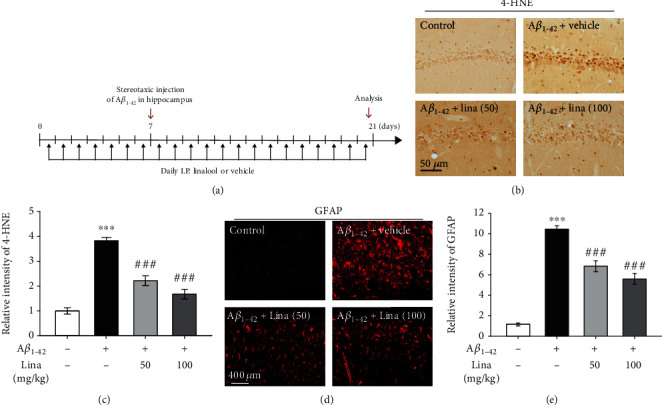
Linalool reduces oxidative stress and gliosis in the hippocampus of A*β*_1-42_-injected rats. (a) Experimental scheme. (b) Representative images of 4-HNE immunostaining, an indicator of lipid peroxidation showing oxidative stress. (c) Quantification of the intensity of 4-HNE immunostaining in the hippocampal CA1 region (*n* = 5). Data are presented as mean ± SEM. ^∗∗∗^*P* < 0.001 vs. vehicle-treated rats, ^###^*P* < 0.001 vs. A*β*_1-42_-injected rats; Tukey's multiple comparison test. (d) Representative images of GFAP immunostaining in hippocampal CA1 region. (e) Quantification of the intensity of GFAP immunostaining in the hippocampal CA1 region (*n* = 5). Data are presented as mean ± SEM. ^∗∗∗^*P* < 0.001 vs. vehicle-treated rats, ^###^*P* < 0.001 vs. A*β*_1-42_-injected rats; Tukey's multiple comparison test. I.P.: intraperitoneal injection; Lina (50): linalool 50 mg/kg I.P.; Lina (100): linalool 100 mg/kg I.P.

To determine whether these finding extend to a mammalian model of AD, the rat unilateral A*β*_1-42_ lesion model of AD was used [26]. A*β*_1-42_ and vehicle were stereotaxically injected into the hippocampus. Linalool was administered intraperitoneally once a day for 7 days before A*β*_1-42_ injection and for 14 days after A*β*_1-42_ injection (Figure 6(a)). To investigate effects of linalool on A*β*_1-42_-induced oxidative damage, we performed immunostaining with an anti-4-HNE antibody in the hippocampal tissues. Immunohistochemical analysis showed that 4-HNE levels were significantly increased in the CA1 layer of the hippocampus of A*β*_1-42_-treated rats compared to vehicle-treated rats (Figures 6(b) and 6(c)). This A*β*_1-42_-induced increase of 4-HNE levels was significantly attenuated at 21 days after linalool treatment (50 and 100 mg/kg/day) (Figures 6(b) and 6(c)). Next, we used a GFAP antibody to examine glial reactivity in the hippocampus of A*β*_1-42_-induced rats. Increased GFAP immunostaining was detected in the hippocampus of A*β*_1-42_-injected rats as compared to the control. Hypertrophic astrocytes were apparent in the hippocampus of the A*β*_1-42_-injected rats, while linalool treatment (50 and 100 mg/kg/day) reduced the number of GFAP+ immunoreactive astrocytes (Figures 6(d) and 6(e)). Taken together, these results provide evidence that linalool can prevent A*β*_1-42_-induced oxidative stress and gliosis in the hippocampus of rats.

## 4. Discussion

In our previous study, we discovered linalool as one of the major monoterpenes secreted in forests and that it has the potential to alleviate the cytotoxicity of A*β* [13]. Based on that, in this study, we investigated how linalool exhibits neuroprotective effects using *Drosophila* models that express human A*β* in neurons or developing eyes as well as an A*β*_1-42_-injected rat model. As a result, we found that linalool suppressed ROS production and inflammatory response induced by A*β* expression and reduced neurodegeneration. These results are consistent with the results of previous studies showing that linalool has antioxidant and anti-inflammatory properties in mouse models and *in vitro* assay [17, 33], which suggests that these properties are the main mechanisms underlying the neuroprotective effect of linalool.

Several previous studies have reported the neuroprotective effect of linalool [20, 34]. In fact, linalool mitigates cognitive impairment and brain damage caused by A*β* in a transgenic AD mouse model [23]. Moreover, linalool decreases the amount of A*β* in the brains of mice, but it is unclear whether linalool decreases A*β* production or increases A*β* clearance [23]. The amount of A*β* accumulation is determined by the sum of A*β* production and A*β* clearance in the triple transgenic AD mouse model expressing PS_1M146V_, tau_P301L_, and APP_swe_. However, the *Drosophila* model used in the present study does not undergo *β*-amyloidosis and directly expresses A*β*42; therefore, in our model, we only observed whether linalool affects A*β* clearance. As linalool did not affect the accumulation and aggregation of A*β*42 in our model, we believe that linalool does not influence A*β* clearance. Herein, linalool seems to inhibit A*β* production rather than its clearance. Further studies are needed to determine how linalool inhibits A*β* production in the future.

In addition, linalool inhibits not only A*β* accumulation but also free radical formation and inflammatory reaction induced by A*β* [23]. However, from these results alone, it is unknown whether linalool inhibits A*β* formation and consequently inhibits free radical formation and inflammatory responses or whether it inhibits A*β* formation while simultaneously inhibiting free radical formation and inflammatory responses. In other words, it is unclear whether the neuroprotective effects of linalool against A*β* toxicity were entirely attributable to A*β* reduction itself or whether the neuroprotective effects were also affected by antioxidant and anti-inflammatory properties of linalool as well as A*β* reduction. However, as A*β* reduction was not observed in our model, our results clearly demonstrate that linalool acts as a neuroprotective agent regardless of A*β* levels. Similar to our results, linalool reduced apoptosis and oxidative stress and attenuated cognitive deficits in a mouse model in which A*β* was directly injected without *β*-amyloidosis [24]. Collectively, linalool is expected to inhibit AD progression through various physiological activities.

We found that linalool protects neurons by reducing free radical production and inflammatory responses caused by A*β*42, thus reducing apoptosis. However, the mechanism through which linalool acted to suppress free radical formation and the inflammatory response induced by A*β*42 was unknown. Linalool appeared to regulate ROS through Nrf2 and p21 and suppress NF-*κ*B expression, which is increased by ROS [35, 36]. Linalool is also reported to prevent mitochondrial apoptosis by inhibiting ROS via suppressing the interaction between SIRT3 and SOD2 [37], suggesting that linalool may prevent the production of free radicals by regulating the interaction between SIRT3 and SOD2 and controlling Nrf2 expression [38]. Additionally, a study reported that linalool alleviates the lipopolysaccharide-stimulated inflammatory response through the activation of the Nrf2/HO-1 pathway in BV2 microglia [32]. Linalool not only enhances the expression of Nrf2 and HO-1 but also suppresses the expression of inflammatory mediators such as TNF-*α*, IL-1*β*, NO, and PGE 2 [32]. Linalool also showed an inhibitory effect on inflammation via the inhibition of NF-*κ*B activity in mice with allergic asthma caused by ovalbumin exposure [39]. Future studies are warranted to confirm whether linalool reduces the A*β*42-stimulated inflammatory response through the regulation of the Nrf2/HO-1 pathway and NF-*κ*B activity. Moreover, changes in the expression levels of inflammatory mediators TNF-*α*, IL-1*β*, and PGE 2 by linalool treatment in AD models should also be addressed.

AD is often accompanied by depression [40], and depression is reported to increase fibrous tangles in AD [41]. Linalool is known to have a beneficial effect on depression, which is one of the main symptoms of AD. In fact, linalool activates GABA_A_ receptors in the brain through smell and reduces depression [42]. When the sense of smell is blocked, linalool cannot activate the GABA_A_ receptor, preventing its antidepressant effect [42]. Further, it was confirmed that there were fewer functional GABA_A_ receptors in the AD brain than in the normal brain [43]. Depression medications have no effect on depression associated with AD, and to date, no medication is available that treats both diseases [44]. However, linalool can suppress depression through the GABA_A_ receptor and AD progression via its antioxidant and anti-inflammatory properties; thus, it may have an effect on AD-related depression.

Recently, many AD drugs have been developed, among which therapeutic agents developed by immunologic approaches targeting A*β* clearing have attracted attention [45, 46]. Among them, both monoclonal antibodies and intravenous immunoglobulin showed significant results in the reduction of A*β* plaques in clinical trials, but the AD patient's cognitive improvement effect was insufficient [47–50]. Therefore, in the treatment of AD, it is possible that therapeutic agents that act on pathological mechanisms, such as inflammatory response and ROS production increased by A*β* in addition to removal of A*β*, may be effective. In particular, for the treatment of multifactorial diseases such as AD, the development of therapeutic agents using natural products such as linalool, which regulates various biological targets, should be considered.

## 5. Conclusions

In conclusion, the results of this study demonstrate that linalool prevents the neurotoxicity of A*β* by inhibiting free radical production and inflammatory response. Therefore, linalool is expected to be used as a preventive or therapeutic agent for AD.

## Data Availability

All data generated or analyzed during this study are included in this published article.
